# Faecal Virome Analysis of Wild Animals from Brazil

**DOI:** 10.3390/v11090803

**Published:** 2019-08-30

**Authors:** Matheus A. Duarte, João M. F. Silva, Clara R. Brito, Danilo S. Teixeira, Fernando L. Melo, Bergmann M. Ribeiro, Tatsuya Nagata, Fabrício S. Campos

**Affiliations:** 1 Faculdade de Agronomia e Veterinária, Universidade de Brasília, Brasília-DF 70.910-900, Brazil; 2 Departamento de Biologia Celular, Instituto de Biologia, Universidade de Brasília, Brasília-DF 70.910-900, Brazil; 3 Núcleo de Atendimento e Pesquisa de Animais Silvestres, Universidade Estadual de Santa Cruz, Ilhéus-BA 45.662-900, Brazil; 4 Departamento de Fitopatologia, Instituto de Biologia, Universidade de Brasília, Brasília-DF 70.910-900, Brazil; 5 Laboratório de Bioinformática e Biotecnologia, Campus de Gurupi, Universidade Federal do Tocantins, Tocantins-TO 77.410-570, Brazil

**Keywords:** Beak and feather disease virus (BFDV), chicken anemia virus (CAV), *Adenoviridae*, Psittacine adenovirus 3, Chapparvovirus, *Gyrovirus*, *Norovirus*, *Smacoviridae*

## Abstract

The Brazilian Cerrado fauna shows very wide diversity and can be a potential viral reservoir. Therefore, the animal’s susceptibility to some virus can serve as early warning signs of potential human virus diseases. Moreover, the wild animal virome of this biome is unknown. Based on this scenario, high-throughput sequencing contributes a robust tool for the identification of known and unknown virus species in this environment. In the present study, faeces samples from cerrado birds (*Psittacara leucophthalmus*, *Amazona aestiva*, and *Sicalis flaveola*) and mammals (*Didelphis albiventris*, *Sapajus libidinosus*, and *Galictis cuja*) were collected at the Veterinary Hospital, University of Brasília. Viral nucleic acid was extracted, submitted to random amplification, and sequenced by Illumina HiSeq platform. The reads were de novo assembled, and the identities of the contigs were evaluated by Blastn and tblastx searches. Most viral contigs analyzed were closely related to bacteriophages. Novel archaeal viruses of the *Smacoviridae* family were detected. Moreover, sequences of members of *Adenoviridae*, *Anelloviridae*, *Circoviridae*, *Caliciviridae*, and *Parvoviridae* families were identified. Complete and nearly complete genomes of known anelloviruses, circoviruses, and parvoviruses were obtained, as well as putative novel species. We demonstrate that the metagenomics approach applied in this work was effective for identification of known and putative new viruses in faeces samples from Brazilian Cerrado fauna.

## 1. Introduction

Cerrado is a Brazilian savannah and one of the most diverse biomes in the world. However, it has been threatened by livestock and agricultural crop production expansion. This fact endangers not just local fauna but also adjacent biomes, such as the Amazon [1]. Associated with wildlife conservation, environmental degradation is a problem for public health, since wild animals can serve as reservoirs or intermediate hosts for new zoonotic pathogens such as viruses. Many zoonotic virus diseases have been emerging or re-emerging, especially, those caused by alphaviruses (*Chikungunya virus*, *Mayaro virus*, *Madariaga virus*) [2,3], bunyaviruses (*Oropouche virus*) [4], and flaviviruses (Zika, dengue, and yellow fever viruses) [5]. In this scenario, the deforestation can increase the contact between humans and animals, inclusively vectors, contributing to the emergence of diseases outbreaks in different regions of the world [6,7].

In Cerrado, the wildlife–livestock–human relationship is a reality [1]. Consequently, zoonoses surveillance is an important measure to control possible emerging diseases. In addition to livestock production, urban expansion brings another concern: the presence of pets, which includes exotic animals and domestic rodents, birds, and pigs. Local fauna has potential to become pathogen reservoirs, especially birds and rodents, that can spread zoonotic and non-zoonotic pathogens to these pets since their domestic counterparts are probably more susceptible to infection by these pathogens [7,8]. In addition, it is well reported the contribution of exotic animals to human diseases, with many examples of pathogens transmission, such as *Salmonella enterica*, *Francisella tularensis*, *Chlamydophila psittaci*, and *Pasteurella multocida* [8,9]. These animals can introduce new species or strains in nature and can harbor local isolates. 

Most emerging diseases are zoonotic [10], so minimizing the contact between wild and non-wild animals is necessary. Epidemiological surveillance with focus in native fauna is a way to identify possible threats to humans and non-human animals that are hidden in reservoirs or that are as yet unknown. To achieve this goal, metagenomics is a powerful tool. It is estimated that there are approximately 1.67 million unknown viruses of key zoonotic viral families in mammal and bird hosts and that 631,000–827,000 of them are potential zoonotic [11]. Considering this information and the occurrence of new zoonotic emerging diseases in Brazil, the aim of this study was to perform a virus metagenomic investigation to identify known and unknown viruses of faecal virome of birds and mammals of Brazilian Cerrado biome.

## 2. Materials and Methods 

### 2.1. Sample Collection

Faecal samples from seven specimens of birds (*n* = 4 *Amazona aestiva*, *n* = 1 *Sicalis flaveola*, and *n* = 2 *Psittacara leucophthalmus*) and three specimens of mammals (*n* = 1 *Didelphis albiventris*, *n* = 1 *Sapajus libidinosus*, and *n* = 1 *Galictis cuja*) were collected early morning from the ground of the animal enclosures and individually placed in sterilized plastic recipients, in the Veterinary Hospital of the University of Brasilia in 2016. The animals showed clinical signs of apathy and were monitored. The samples were transported refrigerated to the Laboratory of Virology of the Cell Biology Department at University of Brasilia and stored in a freezer at −80 °C. 

### 2.2. Viral Enrichment and Nucleic Acid Extraction 

Faecal samples were grouped in two pools—one (Pool 1) with only birds (1- *A*. *aestiva* and *S*. *flaveola*), and another (Pool 2) with mammals and birds (2- *P*. *leucophthalmus*, *D*. *albiventris*, *S*. *libidinosus*, and *G*. *cuja*). They were resuspended and homogenized vigorously in Hanks’s balanced solution and centrifuged at 2500× *g* for 90 min at 4 °C. The supernatant was filtered using a 0.45 μm syringe filter and ultracentrifugated on a 25% sucrose cushion at 190,000× *g* for 4 h at 4 °C. The pellets were resuspended in TE buffer (10 mM Tris pH 7.4; 1 mM EDTA pH 8.0) and treated with 100 U of DNase I (Invitrogen, Carlsbad, EUA) and 20 U of RNase A (Invitrogen, Carlsbad, EUA) at 37 °C for 2 h. The putative viral RNA and DNA present in the resulting sample were extracted using the commercial High Pure Viral Nucleic Acid Kit (Roche, Basel, Switzerland) following the manufacturer’s instructions.

### 2.3. Sequence-Independent Amplification of Viral Nucleic Acids 

Random PCRs were performed prior to the metagenomic sequencing using a particle-associated nucleic acid (PAN-PCR) approach [12]. For the extracted DNA, the first reaction was made in a final volume of 50 μL, containing 5 μL (~500 ng) of template, 0.8 μM of the K-random-s primer (5′-GAC CAT CTA GCG ACC TCC ACM NN MNM-3′), 0.2 mM of each dNTP, 1× PCR buffer, 2.5 mM MgCl_2_, and 1 U of Taq DNA polymerase (Invitrogen, Carlsbad, USA). Amplification PCR condition was: initial denaturation cycle at 94 °C for 3 min, followed by 35 cycles at 94 °C for 50 s, 53 °C for 50 s, and 72 °C for 50 s, and final extension at 72 °C for 3 min. For generating products with the conserved region of the K-random-s primer, an extension reaction was performed using a Klenow fragment DNA polymerase (New England Biolabs, NEB, Ipswich, USA) with 20 µL of template and 5 U of enzyme at 37 °C for 2 h. A third reaction for the amplification of the products was done in a volume of 50 μL, containing 5 μL of template, 0.4 μM of the K-s primer (5’-GAC CAT CTA GCG ACC TCC AC-3′), 0.2 mM of each dNTP, 1 × PCR buffer, 2.5 mM MgCl_2_, and 1 U of Taq DNA polymerase (Thermo-Fisher Scientific, Waltham, EUA). Amplification condition was the same as above. For the extracted RNA, a cDNA synthesis was carried out initially with 10 µL of RNA sample and 2.5 µM of the K-random-s primer incubated at 75 °C for 5 min, followed by a reaction with 200 U of M-MuLV (NEB, Ipswich, USA), 40 U of RNase OUT (Thermo-Fisher Scientific, Waltham, EUA), 0.05 M of DTT, and 1 × M-MuLV buffer incubated at 90 °C for 10 min and at 42 °C for 1 h. Extension was also performed using a Klenow fragment DNA polymerase (NEB, Ipswich, USA) at the same conditions as for the DNA products. Final PCR was made in 50 µL final reaction volume containing 5 μL of cDNA, 0.4 μM of the K-s primer, 0.2 mM of each dNTP, 1 × PCR buffer, 2.5 mM MgCl_2_, and 1 U of Taq DNA polymerase (Thermo-Fisher Scientific, Waltham, EUA). The amplified products were visualized by electrophoresis in a 1% agarose gel and purified with the commercial Illustra GFX PCR DNA and Gel Band Purification Kit (SigmaAldrich, San Luis, USA) following the manufacturer’s instructions.

### 2.4. Metagenomic Sequencing and Bioinformatics 

The purified products were sheared and submitted to library construction using the TrueSeq DNA Nano kit at Macrogen Inc. (Seoul, South Korea). High-throughput sequencing was performed in Illumina HiSeq 2500 platform with 100 nt paired-end. Quality control of the reads was analyzed in FastQC software [13]. Trimming quality and filtering were carried out with BBDuk tool [14] with the removal of the adapters and primer sequences of right and left ends. The reads were de novo assembled in Megahit v1.1.3 [15] and in SPAdes 3.13.0 [16]. The kmer sizes specified were 21, 41, 61, 81, and 99 bases, and 21, 33, 55, 77, and 99, respectively. The contigs were submitted to tblastx search against to the RefSeq Virus database of the NCBI with E-value cutoff of 1e-10. False positives were filtered using blastn search against to the non-redundant (nt) database with cutoff of 1e-20. Reads sequences were deposited in SRA database with the accession number PRJNA556823.

### 2.5. Phylogenetic Analysis 

Eukaryotic viral sequences obtained in this work were deposited in GenBank with the following accession numbers—MN025529, MN025530, MN153802, MN175605, MN175606, MN175607, MN175608, MN175609, MN175610, MN175611, MN175612, MN175613, MN175614, and MN175615—and used to posterior phylogenetic analysis. Phylogenetics analysis was performed for Adenoviridae, Anelloviridae, Circoviridae, Parvoviridae, and Smacoviridae families. Alignment was carried out using the MUSCLE and ClustalW algorithm, and the trees were constructed by Neighbor-Joining (NJ) and Maximum-likelihood (ML) methods in MEGA7 [17], RAxML v8.2 [18] and IQ-TREE v1.6.10 [19] software. The jModelTest v2.1.10 and ProtTest v3.0 tools were used to estimate the best substitution models. Bootstrap was performed with 1000 replicates.

## 3. Results

### 3.1. Pool Information

Samples were divided into two pools. Pool 1 was composed by faecal samples of *A. aestiva* and *S. flaveola* and pool 2 with *P. leucophthalmus*, *D. albiventris*, *S. libidinosus*, and *G. cuja* samples. *A. aestiva* is a psittacine species that has a wide distribution in Brazil and can be found in different natural habitats or as a pet. It occurs also in Argentina, Paraguay, and Bolivia. *P. leucophthalmus*, a psittacine species, extends widely over South America and is common in some urban areas [20]. *S. flaveola* is a passerine found naturally in South America [21]. *D. albiventris* is a marsupial found in Brazil, Paraguay, and Argentina, including urban areas [22]. *S. libidinosus* is a New World monkey endemic to Brazil that was recently found infected with Zika virus. It is found in Cerrado and Caatinga biomes [23,24]. *G. cuja*, a carnivore, is a mustelid with broad distribution over South America [25].

### 3.2. Pool 1

**Figure 1:**
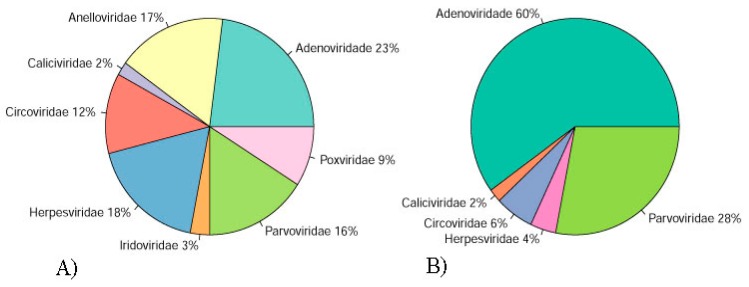
Percentage of the contigs with blast search hits with animal viruses classified in families of pool 1 (**A**) and pool 2 (**B**) assembled using Megahit v1.1.3 and SPAdes 3.13.0 and filtrated by final tblastx.

**Figure 2:**
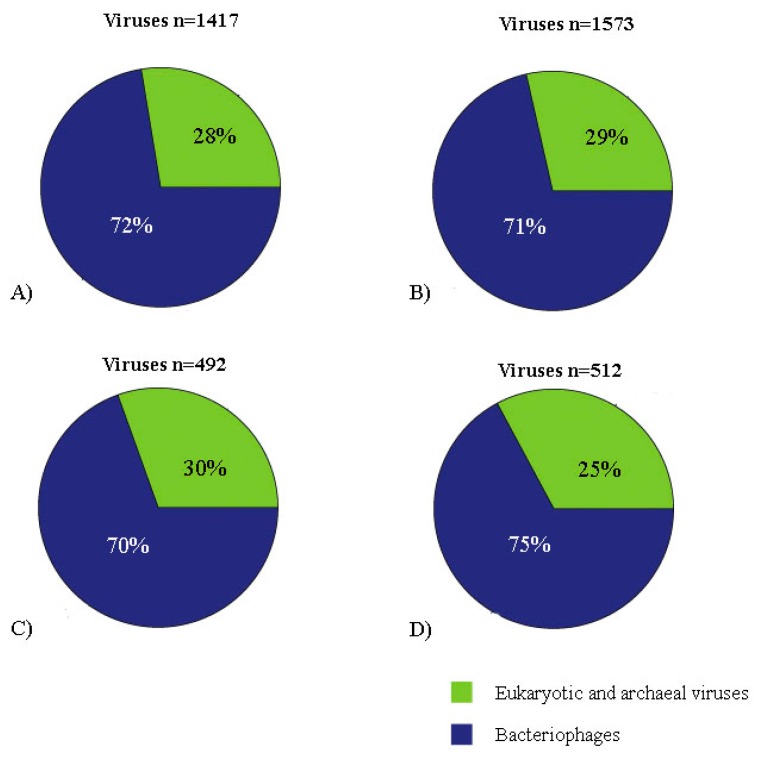
Viral contigs classification (bacteriophages, eukaryotic and archaeal viruses) represented by pie charts. **A**: contigs of pool 1 assembled using Megahit v1.1.3 and obtained by final tblastx filtration (cutoff: 10e-10). **B**: contigs of pool 1 assembled using SPAdes 3.13.0 and obtained by final tblastx filtration (cutoff: 10e-10). **C**: contigs of pool 2 assembled using Megahit v1.1.3 and obtained by final tblastx filtration (cutoff: 10e-10). **D**: contigs of pool 2 assembled using SPAdes 3.13.0 and obtained by final tblastx filtration (cutoff: 10e-10).

Illumina sequencing generated 22,586,752 and 25,049,662 paired-end reads for DNA and RNA samples, respectively. Reads were concatenated in a single archive. Final number of trimmed reads was 47,226,100. The contigs were de novo assembled using Megahit v1.1.3 and SPAdes 3.13.0 [15,16]. Megahit generated 22,986 contigs with average length of 813 nt and standard deviation of 2043. Minimum and maximum contig lengths were 200 nt and 222,414 nt. SPAdes produced 27,311 contigs with average length of 674 nt and standard deviation of 1003. Minimum and maximum contig lengths were 100 nt and 59,423 nt. Contigs of both assemblers were concatenated and submitted to tblastx search against RefSeq virus database and later to Blastn search against to the nt database. Contig with blast search hits with animal viruses is represented in Figure 1A and Figure 2A,B. A list of eukaryotic viral contigs with significant tblastx hits and their GenBank accession numbers are shown in Table S1.

### 3.3. Pool 2

Illumina sequencing generated 22,992,728 and 28,478,188 paired-end reads for DNA and RNA samples. Reads were concatenated in a single archive. Final number of trimmed reads was 51,332,426. The contigs were de novo assembled using Megahit v1.1.3 and SPAdes 3.13.0 [15,16]. Megahit generated 2642 contigs with average length of 2113 nt and standard deviation of 5683. Minimum and maximum contig lengths were 200 nt and 80,761 nt. SPAdes produced 4139 contigs with average length of 1450 nt and standard deviation of 3785. Minimum and maximum contig lengths were 100 nt and 62,492 nt. The concatenated contigs were submitted to tblastx search against RefSeq virus database and later to Blastn search against to the Nucleotide database. Viral contigs classification is represented in Figure 1B and Figure 2C,D. A list of eukaryotic viral contigs with significant tblastx hits, and their GenBank accession numbers are shown in Table S2. 

### 3.4. Adenoviridae

**Figure 3:**
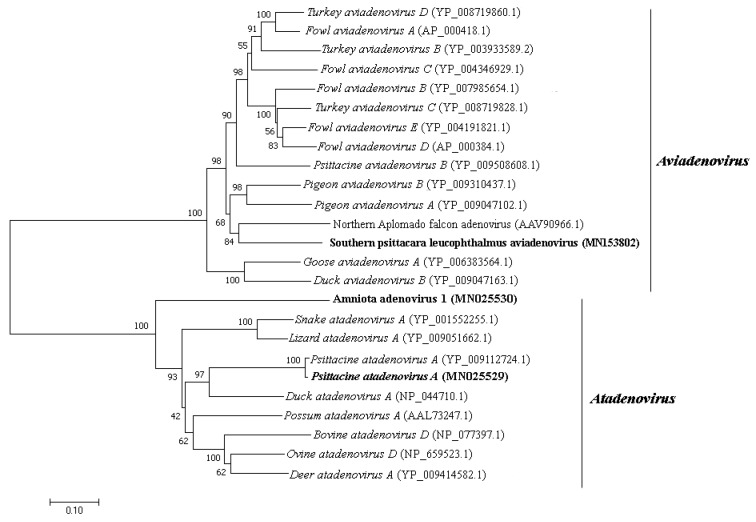
Neighbor-joining tree based on hexon protein amino acid sequence of (~900 aa) 25 aviadenovirus and atadenovirus sequences. The tree is midpoint rooted and was built in MEGA7 software using Jones-Taylor-Thornton (JTT) substitution-rate matrix with gamma distribution (+G) in accordance to ProtTest v3.0 analysis. Alpha shape parameter was estimated, and bootstrap was performed with 1000 replicates. Adenovirus sequences identified in this study are labeled in bold type. GenBank accession numbers of the viral sequences are shown in parentheses.

**Figure 4:**
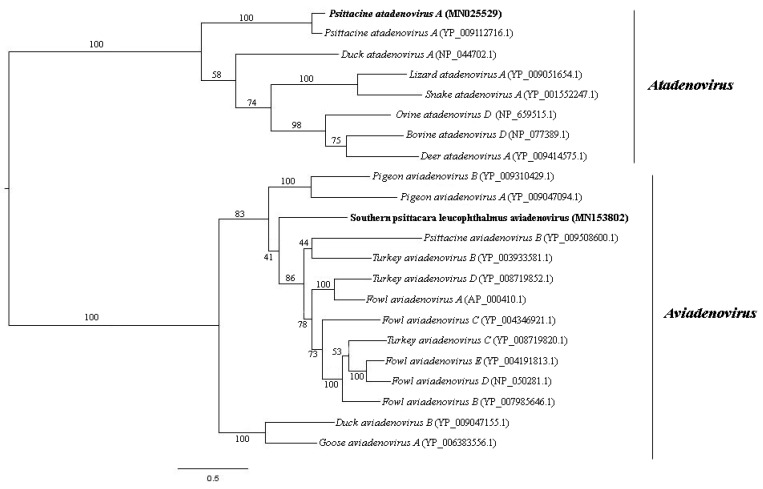
Maximum-likelihood tree based on DNA polymerase protein amino acid sequence (~1300 aa) of 22 aviadenovirus and atadenovirus sequences. The tree is midpoint rooted and was built in RAxML v8.2 software using Le and Gascuel (LG) substitution-rate matrix with gamma distribution (+G) and invariant sites (+I) in accordance to ProtTest analysis. Bootstrap was performed with 1000 replicates. Adenovirus sequences identified in this study are labeled in bold type. GenBank accession numbers of the viral sequences are shown in parentheses.

**Figure 5:**
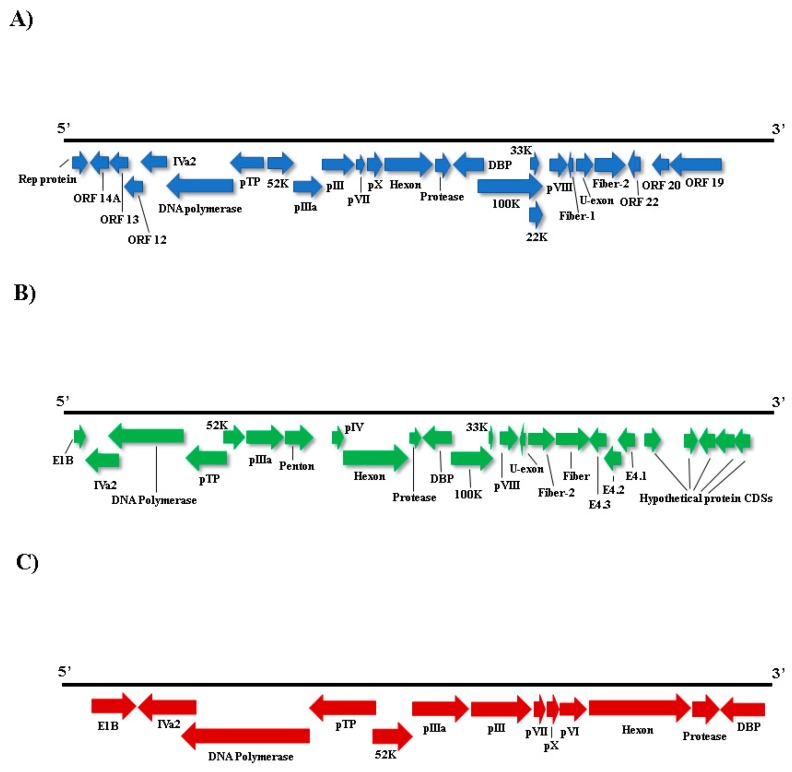
Schematic genome representation of three adenoviruses. **A**: in blue, a possible new adenovirus, the proposed southern psittacara leucophthalmus aviadenovirus with nearly complete genome obtained (~35 kb). **B**: in green, nearly complete viral genome sequence of the proposed psittacine adenovirus 3 isolate BR_DF (~30 kb). **C**: in red, a putative new species with partial genome, the proposed amniota adenovirus 1 (~18 kb).

*Adenoviridae* is a family of non-enveloped dsDNA viruses with non-segmented linear genome of 26–48 kilo-base pair (kb or kbp) in size. It is currently divided into five genera [26]. They are involved in many respiratory and gastrointestinal animal diseases and are included in surveillance programs given their importance in public health [27,28]. Adenovirus-like sequences close to *Aviadenovirus* and *Atadenovirus* genera were detected in pool 1. The same genera were detected in pool 2 besides *Mastedonovirus*. It is the viral family from both samples with the greatest number of viral contigs obtained. Amino acid identity ranges from 32.9% to 92.7% for pool 1, with contig length varying from 266 to 2606 nt, and 42.1% to 100% for pool 2, with contig length varying from 115 to 20,267 nt. Phylogenetic analyses were performed using DNA polymerase and hexon amino acid sequences of aviadenoviruses and atadenoviruses obtained from pool 2, including the most closely related sequences identified by tblastx search (Figure 3 and Figure 4). For hexon amino acid sequence, pairwise identity between contig NODE 39 and Northern Aplomado falcon adenovirus (AAV90966.1) was 73.9%. For contig k119 2350 and psittacine adenovirus 3 (*Psittacine atadenovirus A*) (YP_009112724.1), 97.6%. For contig k119 1050 and *Duck atadenovirus A* (NP_044710.1), 64.5%. DNA polymerase amino acid sequence pairwise shows identity of 90.2% between contig k119 2155 and psittacine adenovirus 3 (YP009112716.1). For contig k119 380 and *Fowl aviadenovirus A* (AP_000410.1), 61.7% of amino acid identity. For contig k119 1050 and *Duck atadenovirus A* (NP_044710.1), 49.1% (Table 1). Schematic genome representation of three novel putative adenovirus species is shown (Figure 5). 

### 3.5. Anelloviridae

**Figure 6:**
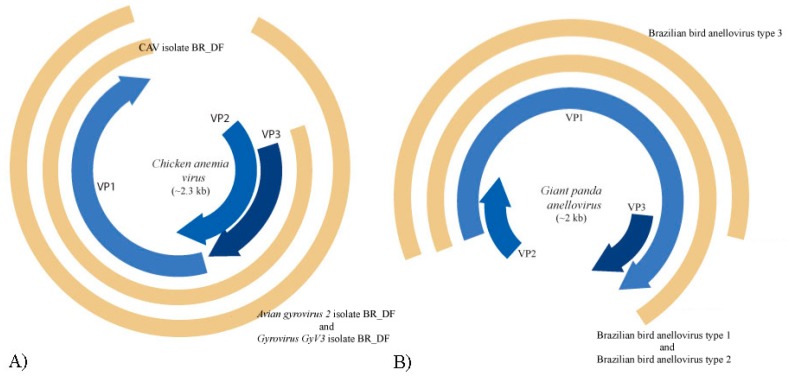
Genome representation of known and novel anelloviruses. **A**: In blue, ORFs VP1, VP2 and VP3 of the prototypic gyrovirus, chicken anemia virus (CAV), and, in brown, the proposed CAV isolate BR_DF (contig k199 16753), avian gyrovirus 2 isolate BR_DF (contig k119 6992) and gyrovirus GyV3 isolate BR_DF (contig k119 6843). **B**: In brown, the proposed Brazilian bird anellovirus type 1, type 2 and type 3 (contigs NODE 177, NODE 986 and NODE 1090, respectively) and, in blue, ORFs VP1, VP2, and VP3 of the closely related giant panda anellovirus.

**Figure 7:**
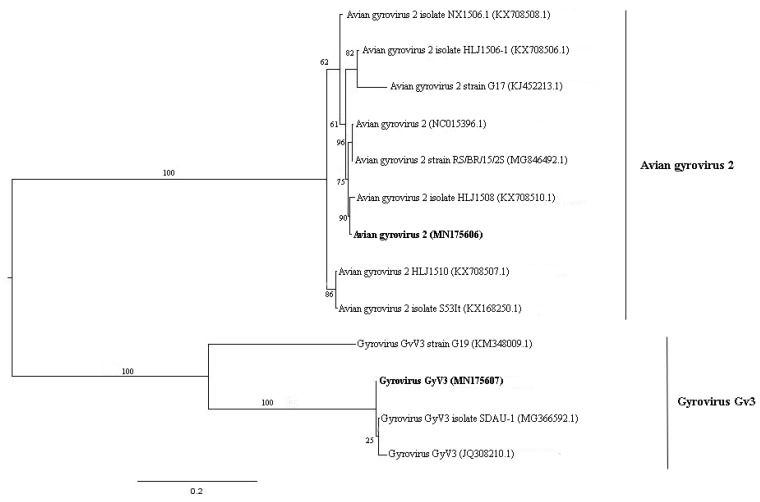
Maximum-likelihood tree based on ORF 1 nucleotide sequence (~1.4 kb) of 13 avian gyrovirus 2 and gyrovirus GyV3 sequences. The tree is midpoint rooted and was built in RAxML v8.2 software using general time-reversible (GTR) substitution model with gamma distribution (+G) in accordance to jModelTest v2.1.10 analysis. Bootstrap was performed with 1000 replicates. Anellovirus sequences identified in this study are labeled in bold type. GenBank accession numbers of the viral sequences are shown in parentheses.

**Figure 8:**
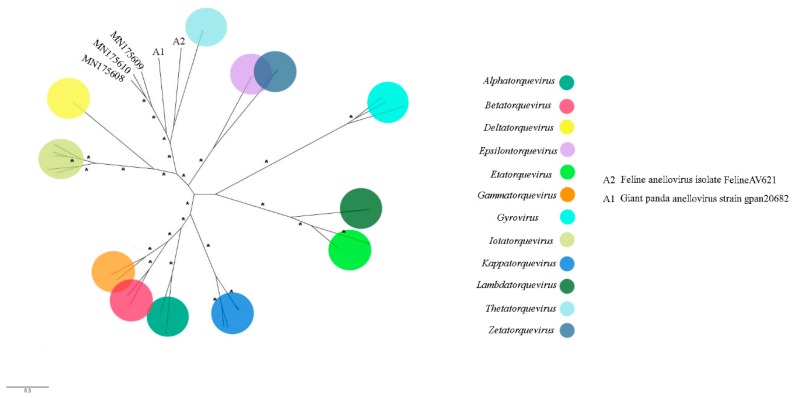
Maximum-likelihood tree based on ORF 1 nucleotide sequence (~2.3 kb) of 32 anellovirus. The tree is unrooted and was built in IQ-TREE v1.6.10 software using transversion substitution model (TVM) with gamma distribution (+G) and invariant sites (+I) in accordance to jModelTest v2.1.10 analysis. Bootstrap was performed with 1000 replicates, and values equal to 70 or more are represented by asterisks. GenBank accession numbers of the anellovirus sequences identified in this study are shown. Details about the sequences of the phylogenetic tree are shown in Table S3.

Anelloviruses are non-enveloped viruses with negative sense and circular ssDNA genome with 2.1–3.9 kb in size. They have a wide distribution in human population and were found in different vertebrate species, including birds and mammals [29]. Anellovirus-like sequences are present only in pool 1. Most contigs were related to ORF1 of Seal anellovirus 4 after tblastx search. Their amino acid identity varied from 33.3% to 52.5% and sequence length from 425 to 2659 nt. Contig sequences closer to giant panda anellovirus, *Torque teno canis virus* and *Torque teno sus virus k2b*, showed amino acid identities of 34.4%, 36.0% to 39.8%, and 52.5%, with lengths of 882 nt, 962 to 1250 nt, and 555 nt, respectively. *Chicken anemia virus*, avian gyrovirus 2, and gyrovirus GyV3 species were also detected (Figure 6A). ORF1 nucleotide sequence was used for phylogenetic analyses since it is used as species demarcation criteria (Figure 7 and Figure 8). Between chicken anemia virus isolate (contig k199 16753) and closely related isolate strain CL37 (JQ308213.1), nucleotide identity was 98.8%. Nucleotide identity between contig k119 6992 and most closely related avian gyrovirus 2 isolate (KX708510.1) was 98.8%. Between contig k119 6843 and most closely related gyrovirus GyV3 (MG366592.1) was 99.4%. For contigs NODE 177, NODE 986 and NODE 1090, identity with giant panda anellovirus (MF327552.1) was 54.6%, 50.7% and 51.7% respectively (Figure 6B) (Table 2). A phylogenetic tree was constructed including just gyroviruses sequences. Another phylogenetic tree was built with the main genera of *Anelloviridae* family and unclassified closely related anelloviruses.

### 3.6. Caliciviridae 

**Figure 9:**
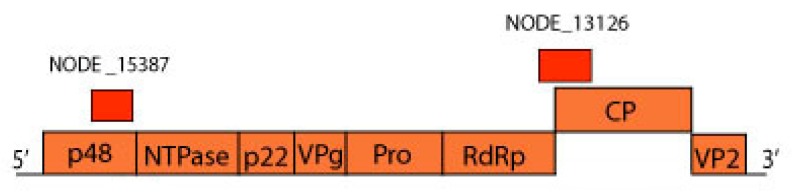
Norovirus genome representation with contigs NODE 15387 (MN175617) and NODE 13126 (MN175616) of pool 1 aligned to ORF1 and ORF2, that encode a polyprotein and capsid protein, respectively.

*Caliciviridae* is a viral family composed of 11 genera of small non-enveloped viruses with non-segmented, linear, positive-sense ssRNA genome that ranges in size from 7.3–8.3 kb. Important animal pathogens that cause enteric and respiratory diseases are included in this family [30,31]. Few calicivirus-like sequences were identified in pools 1 and 2. All the contigs were closely related to *Norovirus* genus, specifically to norovirus GII and GI for pool 1 and norovirus GI for pool 2. Amino acid identity ranged from 70.1% to 96.3 % and 79.7% to 80.3%, with small contigs length of 419 to 501 and 441 to 447 nt, respectively (Figure 9). VP1 and VP2 amino acid sequences were not included in the phylogenetic analyses given the small contig length obtained. 

### 3.7. Circoviridae 

**Figure 10:**
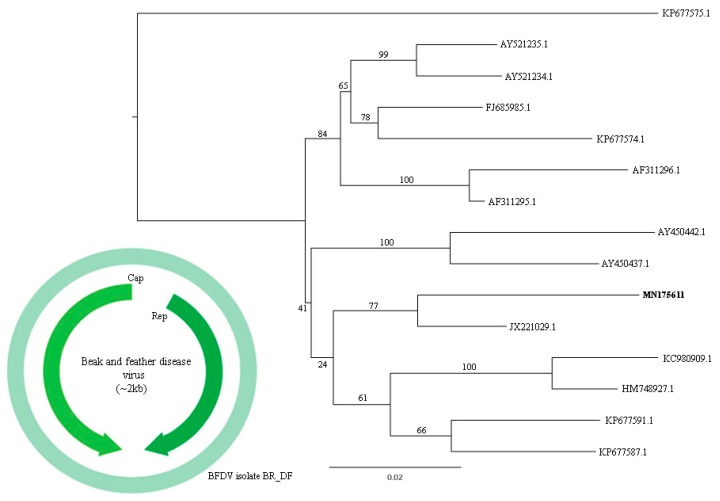
Maximum-likelihood tree based on whole genome nucleotide sequence (~2 kb) of 15 BFDV sequences. The tree is midpoint rooted and was built in IQ-TREE v1.6.10 software using Tamura-Nei nucleotide substitution model (TrN) with gamma distribution (+G) and invariant sites (+I) in accordance to jModelTest v2.1.10 analysis. Bootstrap was performed with 1000 replicates. Circovirus sequence identified in this study are labeled in bold type. GenBank accession numbers of the viral sequences are shown. In light green, the first complete genome sequence of a BFDV Brazilian isolate (MN175611), with capsid and replication proteins represented.

Recently submitted to taxonomic revision, circoviruses are small, non-enveloped viruses with circular ssDNA genome ranging between 1.7–2.3 kb in size that belong to the circular rep-encoding single-strand (CRESS) DNA virus group [32]. Vertebrate and invertebrate hosts have been described for these viruses, affecting especially avian and swine with the smallest known animal viral pathogens included in this group [33,34]. Circovirus-like sequences were detected in both pools and are closely related to *Circovirus* genus, specifically to beak and feather disease virus (BFDV). Partial and complete genome sequences were obtained. Amino acid identity ranged from 73.1% to 96.5% and contigs length between 308 and 1999 nt. Phylogenetic analyses were performed considering genome-wide pairwise identities as demarcation threshold in the group. Nucleotide identity between the isolate BR_DF (contig k199 22721), from the present study, and closest isolate, BFDV-U_PL-543_2008 (JX221029.1), was 94.9 % (Figure 10).

### 3.8. Parvoviridae

**Figure 11:**
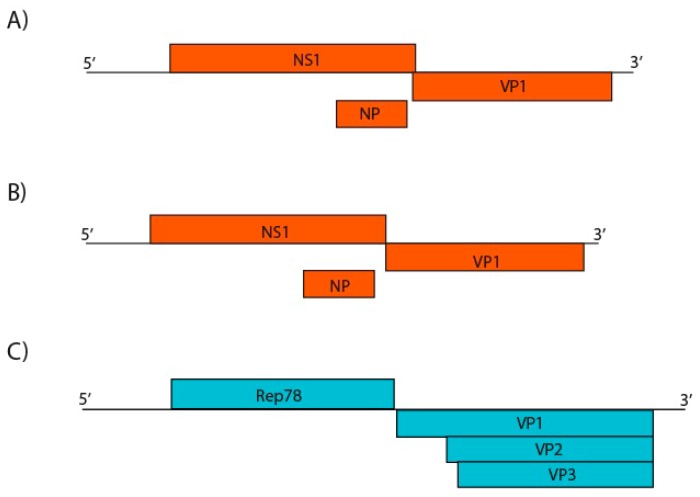
Genome representation of three putative novel parvoviruses identified. **A**: Proposed avian chapparvovirus (contig k119 1463) with conserved NS1 and VP1 sequences, and putative NP (4425 nt). **B**: Proposed psittacara leucophthalmus chapparvovirus (contig k119 15398) species (4116 nt). **C**: Proposed avian adeno-associated virus isolate BR_DF (contig k119 1997) with non- and structural protein sequences (4642 nt).

**Figure 12:**
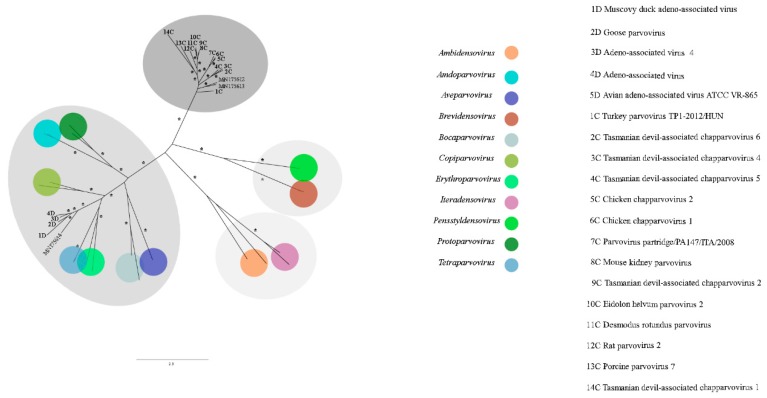
Maximum-likelihood tree based on non-structural protein 1 (NS1) amino acid sequence (~800 aa) of 44 parvoviruses. The tree is unrooted and was built in IQ-TREE v1.6.10 software using rtREV substitution-rate matrix with gamma distribution (+G), invariant sites (+I) and empirical amino acid frequency (+F) in accordance to ProtTest analysis. Bootstrap was performed with 1000 replicates and values equal to 70 or more are represented by asterisks. Chapparvoviruses are in darker grey. *Densovirinae* and *Parvovirinae* subfamilies in light and less dark grey respectively. GenBank accession numbers of the parvovirus sequences identified in this study are shown. Details about the sequences of the phylogenetic tree are shown in Table S3.

*Parvoviridae* is a viral family of small non-enveloped ssDNA viruses with non-segmented and linear genome of 4–6.3 kb in size, involved in many clinical and subclinical animal infections. It is divided into subfamilies *Densovirinae*, found infecting arthropods, and *Parvovirinae*, that infect vertebrates [35,36]. For pool 1, the contigs length ranged from 319 to 4116 nt, showing amino acid identity from 28.9% to 84.5% with other parvoviruses. For pool 2, length varied from 120 to 4425 nt and amino acid identity from 44.0% to 84.8%. Viruses related to both subfamilies were present in pool 1 and are closely related to *Ambidensovirus*, *Iteradensovirus*, *Dependoparvovirus*, and Chapparvovirus genera. For pool 2, just viruses closely related to *Parvovirinae* (*Dependoparvovirus* and Chapparvovirus) were detected. Three nearly complete genome sequences were obtained (Figure 11). The conserved NS1 protein amino acid sequence is a demarcation criterion for the group and was used for phylogenetic analyses (Figure 12). The contigs k119 1463 and k119 15398 showed amino acid identity of 45.3% and 44.9% to turkey parvovirus TP1-2012/HUN (AHF54687.1), respectively. The contig k119 1997 and adeno-associated virus (YP_009552823.1) showed 42.7% amino acid identity. On the other hand, NS1 amino acid identity between k199 1463 and k119 15398 was 56.0% (Table 3). 

### 3.9. Smacoviridae

**Figure 13:**
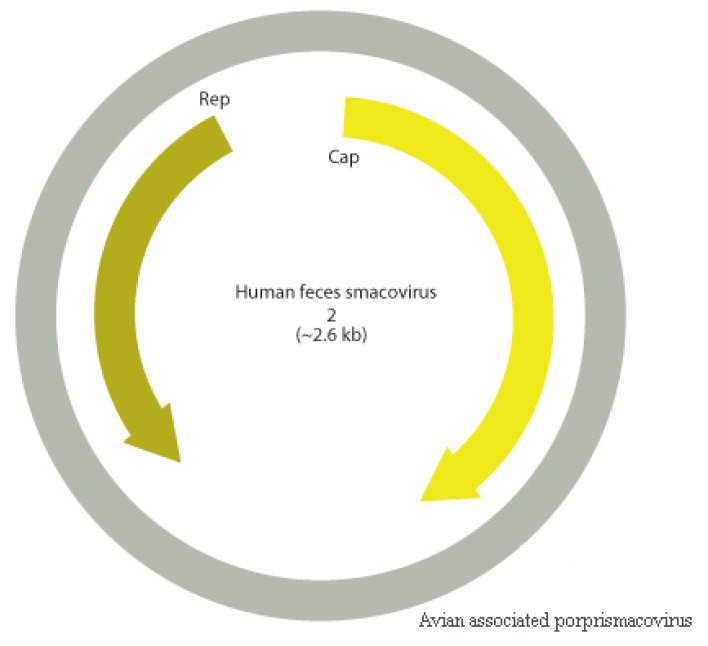
Novel smacovirus, the proposed Avian associated porprismacovirus (MN175615), represented in grey with Human feces smacovirus *2* as prototype for comparison.

**Figure 14:**
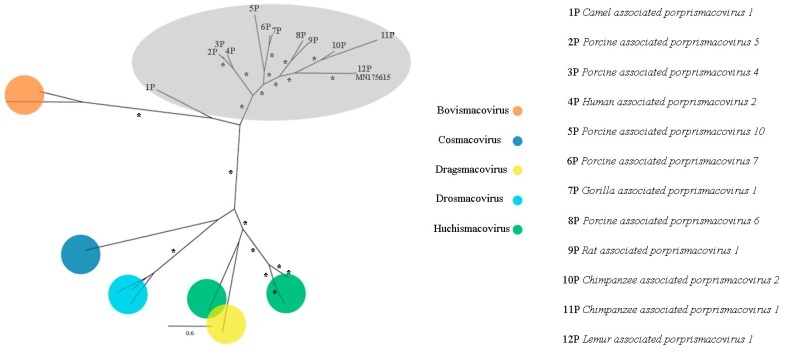
Maximum-likelihood tree based on replication-associated protein (rep) amino acid sequence (~306 aa) of 28 smacovirus sequences. The tree is midpoint rooted and was built in RAxML v8.2 software using Le and Gascuel (LG) substitution-rate matrix with gamma distribution (+G), invariant sites (+I), and empirical amino acid frequency (+F) in accordance to ProtTest analysis. Bootstrap was performed with 1,000 replicates and values equal to 70 or more are represented by asterisks. Nodes in grey area belongs to *Porprismacovirus* genus. GenBank accession number of the proposed Avian associated porprismacovirus identified in this study is shown. Details about the sequences of the phylogenetic tree are shown in Table S3.

Accepted very recently by ICTV, smacoviruses are a group of CRESS viruses with genomes ranging from 2.3–2.9 kb. They were identified by metagenomics in vertebrate faeces and insects and, so far, are not related to any animal disease. At present, the family *Smacoviridae* is divided into six genera [37]. Smacovirus-like sequences were found just in pool 1. All of them were closely related to *Porprismacovirus* genus, in which possible hosts include mammals and birds. Contigs length varied from 620 to 3091 nt and replication-associated protein (rep) amino acid identity with other smacoviruses ranged from 57.8% to 90.3%. Genome-wide identity between contig NODE 726 (MN175615) and the closest smacovirus, *Lemur associated porprismacovirus* 1 isolate SF5 (NC_026320.1), was 67.3% (Figure 13). Phylogenetic analyses were performed using genome-wide and rep amino acid sequence, since the capsid protein (CP) and the replication protein have different evolutionary histories due recombination in the family (Figure 14).

## 4. Discussion

We applied a high-throughput sequencing method to investigate the faecal virome of specimens of wild animals of Cerrado biome, birds (*Amazona aestiva*, *Psittacara leucophthalmus*, and *Sicalis flaveola*) and mammals (*Didelphis albiventris, Sapajus libidinosus*, and *Galictis cuja*). Birds are considered important reservoir hosts of emerging viruses. At least, new 73 viruses were discovered between 2012 and 2014 in this group of animals [38]. Until 2017, these novel described viruses were documented mainly in wild birds, with *Poxviridae*, *Herpesviridae*, and *Adenoviridae* as the most reported DNA virus families with veterinary importance [39]. Regarding to mammals, many investigations focus mainly on bats species [40,41]. Canids [42] and rodents [43] are other groups commonly investigated. In the present study, members of *Adenoviridae*, *Anelloviridae*, *Circoviridae*, *Caliciviridae*, and *Parvoviridae* families were identified. *Aviadenovirus* and *Atadenovirus* sequences were detected in pool 2. Phylogenetic distance of DNA polymerase amino acid sequences greater than 5–15% is one of the several species demarcation criteria in these genera [44]. Considering those criteria, some of our newly identified viruses may merit establishing a novel species for them. By phylogenetic analyses, contig k199 2155 was grouped with psittacine adenovirus 3 (*Psittacine atadenovirus A*) (Figure 4), showing 90.2% of amino acid identity. Hexon sequence in contig k119 2350 also showed high amino acid identity (97.2%) with psittacine adenovirus 3 and was also grouped together (Figure 3). Therefore, these sequences, contigs k199 2155 and k199 2350, represent a new isolate of this virus, with nearly complete genome from *P. leucophthalmus* (Figure 5B). The first description of psittacine adenovirus 3 was in 2014 in an outbreak of avian chlamydiosis and human psittacosis in Hong Kong. In this occasion, it was supposed that this adenovirus caused an immunosuppression that favored *Chlamydophila psittaci* infection in *Amazona farinosa* parrots, resulting in transmission to humans [45]. No other identification was documented since then. 

Low amino acid identity of one hexon sequence in contig k119 1050 indicates a putative novel adenovirus species more closely related to duck adenovirus 1 (DAdV-1) (Figure 3 and Figure 5C), the proposed amniota adenovirus 1. DAdV-1, is the etiologic agent of egg drop syndrome in gallinaceous birds, a disease of great economic importance [46]. DNA polymerase amino acid pairwise identity of contig k119 380 with the closest adenovirus identified, *Fowl aviadenovirus A* (FAdV-1) strain CELO, suggests the presence of a novel species in *P. leucophthalmus* that we tentatively named southern psittacara leucophthalmus adenovirus. Probably this species is closer to Falcon adenovirus 1 (FaAdV-1) analyzing hexon amino acid sequence (contig NODE 39), however no DNA polymerase sequence of FaAdV-1 is available. This virus, first detected in *Falco femoralis septentrionalis,* is involved in severe infectious disease in falcons, characterized by hepatitis, splenomegaly, and enteritis [47]. Further investigation is necessary to evaluate pathogenic potential of this new virus [48,49]. 

Anellovirus-like sequences were also detected. Species and genus demarcation criteria in *Anelloviridae* are based on ORF1 nucleotide sequence identity with cut-off values, respectively, of the 35% and 56% [50]. Three known anellovirus species were detected in pool 1, specifically belonging to the genus *Gyrovirus.* This genus was recently reassigned from *Circoviridae* to *Anelloviridae* considering genomic features of this group [51]. Until 2011, chicken anemia virus (CAV) was the only *Gyrovirus* member identified. From that moment, several novel gyroviruses were characterized in humans and birds [52,53,54]. Partial genome of CAV was found in the present study. This virus is responsible for economic losses in poultry industry since it has tropism for bone marrow-derived cells, causing anemia and immunosuppression [55]. It was thought that CAV has chickens as only natural host, although antibodies in *Coturnix japonica* were detected [56]. Thus far, no other domestic or wild bird was associated to this virus [57]. This is the first report to characterize the presence of CAV in wild birds. By maximum-likelihood and neighbor joining analyses, this novel isolate is most closely related to Brazilian isolates (personal observation). CAV is also reported in mouse, dog, cat, and human faeces, besides human blood [58,59]. However, pathogenesis was not determined for these species. Nearly complete genome of avian gyrovirus 2 (AGV2) was obtained. AGV2 was the second member of *Gyrovirus* described and was first found in sick chickens in the south region of Brazil [60]. It was also identified in chickens with neurologic symptoms in South Africa and involved in infections of healthy people and transplant and HIV-positive patients [61,62]. This is a virus with worldwide distribution and with potential pathogenic importance [61,62,63]. The present isolate belongs to group A and is more closely related to the Chinese isolate HLJ1508 than Brazilian isolates, suggesting different origins of AGV2 in Brazil (Figure 7) [63]. AGV2 was also identified in chickens, ferrets and humans [60,62,64]. This is the first description of this virus in wild birds. Nearly complete genome of gyrovirus GyV3 was obtained (Figure 6A). This species was described in humans in Chile, in chickens in China and in ferrets in Hungary, all showing signs of disease [52,64,65]. This is the first report of GyV3 in Brazil. The present isolate is phylogenetically closer to the Chinese isolate SDAU-1. A possible association is supposed between this virus and the transmissible viral proventriculitis (TVP) disease in chickens and diarrhea in children [52,64,65]. Additionally, ORF1 nucleotide sequence of contigs NODE 117, NODE 986 and NODE 1090 showed low identity to the closest viral species, giant panda anellovirus (GpAV), and between them. Therefore, three probable novel anelloviruses species were identified in this study, the proposed brazilian bird anellovirus type 1, brazilian bird anellovirus type 2, and brazilian bird anellovirus type 3, respectively. Considering the expected genome size of the group, it is probably that contigs NODE 117 and NODE 986 sequences represents nearly complete genomes (Figure 6B). These viruses are related to a clade of unassigned members that includes GpAV, and feline anellovirus strain FelineAV621 [66,67,68] (Figure 8). Since the contigs were obtained from pool1, all the anellovirus sequences have origin in a neognath bird.

*Circoviridade* was other viral family identified. The cut-off criterium for species demarcation in this family is 80 % complete genome nucleotide sequence identity [44]. BFDV was identified in pool 1, with *A. aestiva* as probable host. BFDV is responsible for a common and fatal disease in psittacines characterized by symmetric and progressive feather dystrophy and beak deformities, with no commercial vaccine available [69,70]. In its acute state, it has a high mortality rate, and, in chronic form, birds usually die by secondary infection caused by viral immunosuppression [71]. It has worldwide distribution and is a threat to psittacine conservation and the market of wild birds, especially in Brazil, that harbors great biodiversity of these species [72]. Although the first report in the country dates to 1998, there is few epidemiologic information about circulating viral isolates in the country [73]. Besides, BFDV shows higher mutation rate than other DNA viruses, which rate is similar to RNA viruses. In addition, the recombination is frequent in this species, specifically in the 3’ end of the Cap gene and intergenic region. These mechanisms can explain host diversity and help to support that probably all psittacines can be infected [33]. The present study describes the first complete genome sequence of a BFDV isolate in Brazil. Phylogenetic analysis, performed by clusters using CD-HIT 4.8.1 [74], indicates that the Brazilian isolate is closer to the Poland isolate, BFDV-U_PL-543_2008, with no recombination event detected using RDP4 v.4.96 (Figure 10) [75]. Additionally, a point mutation was observed that changes a cytosine (C) to a thymine (T) and drives an ochre stop codon, producing a truncated capsid protein. Further investigation is necessary to explain the evolutionary history of BFDV in *A. aestiva* host.

Noroviruses-like sequences were found. Members of genus *Norovirus* are especially known to cause gastroenteritis in humans and other hosts. Based on VP1 amino acid sequence, this genus is divided in seven genogroups [76]. GI, GII, GIV, and GVI infect humans, with just GI infecting solely this group [77,78]. In GII, there are viruses able to infect pigs [79]. In GIV, dogs, cats, and lions are hosts [80,81,82]. In GVI, dogs are infected [83]. Another genogroups, GIII, GV, and GVII, are thus far only associated to non-human animals, specifically ruminants (bovines and ovines), murines, and dogs, respectively [84]. Identification of noroviruses in animals raises concern about their zoonotic potential. However, cross-transmission between animals and humans has not been documented. Some evidences support human norovirus (HuNoV) infection in dogs based on the ability of virus attachment to the histo-blood group antigens (HBGAs) receptor and the presence of HuNoV-specific antibodies in these animals, although it was not assigned any clinical disease. Recently, HuNoV GII was identified in wild birds, raising the possibility of these animals being involved in virus transmission [31]. Contigs of small length in pool 1 showed identity to norovirus GII and norovirus GI (Figure 9). However, due to their small size, we could not confirm which viral genogroups were present in our samples. This is the second report of putative noroviruses in birds documented, suggesting these animals as potential reservoirs [31].

Contigs with nearly complete or complete genome sequences from three putative novel species of the family *Parvoviridae* were obtained. Two of them belongs to Chapparvovirus, a novel genus but not recognized by ICTV, so far. The first species of this group identified, *Eidolon helvum parvovirus 2* (EhPV-2), was found in throat swabs of *Eidolon helvum* fruit bats in Africa in 2013, but the genus was proposed just in 2017 with *Porcine parvovirus 7* (PPV7) identification in lung tissues of pigs in China [85,86]. Chapparvoviruses were also found in turkey, rat, Tasmanian devil, chicken, red-crowned crane, and mice faeces, rectal swab of pigs, in grey partridges, in *Desmodus rotundus* kidneys, and in faeces of animals of the present study [87,88,89,90,91,92,93,94]. Screening whole-genome shotgun (WGS) sequences assemblies, chapparvovirus endogenous viral elements (EVE) were identified in vertebrates and more recently in invertebrates [94,95]. This shows that Chapparvovirus has a wide range of host species and supports that vertebrate parvoviruses are not monophyletic as was commonly thought. Besides, this genus includes potential pathogens such as the mouse kidney parvovirus (MKPV), which was associated to chronic nephropathy, raising concern about the involvement of other chapparvoviruses in diseases [96]. NS1 amino acid sequence identity is used as demarcation criteria for genus and species in *Parvoviridae* family, with 30% identity as threshold to novel genus and 95.0% to species [44]. Thus, in the present study, contigs k119 15398 and k119 1463 represent sequences from putative novel viral species, with the proposed names for psittacara leucophthalmus chapparvovirus and avian chapparvovirus, respectively (Figure 11). Contig k119 1463 was found in pool 1, therefore it is associated to a neognath bird host. Contig k119 15398 was identified in pool 2 and, by phylogenetics analyses, was grouped more closely to k119 1463, suggesting that a bird host, probably *P. leucophthalmus*, harbors this species. Basal position of bird infecting chapparvoviruses can mean that a possible transmission between vertebrates and arthropods occurred initially in this group. The other putative novel parvovirus, the avian adeno-associated virus isolate BR_DF species (contig k119 1997), identified in pool 1, belongs to *Dependoparvovirus* genus, that includes viruses that infect vertebrates, but replication in the cell usually depends on another virus, called helper, commonly adenoviruses, herpesviruses or papillomaviruses [97]. In the absence of the helper virus, the cell is nonpermissive and latent infection is established with viral genome integration. Generally, dependoparvoviruses are not pathogenic and are used as vectors for gene therapy (Figure 12) [98]. This novel species is closer to adeno-associated viruses of birds supporting that a neognath bird is the host. Also, some contigs closely related to viral sequences of *Densovirinae* subfamily were obtained due to the feeding habits of these animals.

CRESS viruses are a group of circular ssDNA viruses with a common origin that encode a replication initiator protein (rep). *Smacoviridae* is one of the new families of the group that was recognized by ICTV in 2018 and were thought until recently to have animals as possible hosts since all isolates were identified in faeces or in abdominal of dragonflies by metagenomics analyses [37]. However, CRISPR analysis of *Candidatus* Methanomassiliicoccus intestinalis identified smacovirus originated sequences, which suggests that the host of smacoviruses are most likely archaea [99]. Analyses considering amber codon usage also support this hypothesis. Species and genus criteria demarcation of *Smacoviridae* are based on genome-wide and rep amino acid sequences with cut-off of 77.0% and 40.0%, respectively. The low nearly complete genome sequence identity of contig NODE 726 with the closest smacovirus identified (67.3%), *Lemur associated porprismacovirus 1* isolate SF5 suggests the presence of a novel species in pool 1, which belongs to the *Porprismacovirus* genus also analyzing pairwise amino acid identity of rep (97.2%), the proposed avian associated porprismacovirus, that has a neognath bird as probable host (Figure 13 and Figure 14).

Brazilian fauna has wide diversity, but the animal virome is little explored. The present study was able to identify known animal adenoviruses, anelloviruses, and circovirus. Also, novel putative species of adenovirus, anellovirus, parvovirus, and smacovirus were found. Most sequences obtained belong to non-enveloped ssDNA viruses with small genome (*Anelloviridae*, *Circoviridae*, and *Parvoviridae*). This is in accordance to other metagenomic investigations of faecal viromes [100,101,102]. Additionally, high-throughput sequencing using Illumina HiSeq 2500 platform with 100 nt paired-end allowed the identification of not only complete or nearly complete small genomes but also relatively bigger genomes, as observed for *Adenoviridade*. Some genomes were obtained in singles contigs. However, regarding to RNA viruses, only calicivirus-like sequences were detected. This viral diversity was characterized despite of the small number of animals sampled and shows how wild animals have a complexity and little-known viral microbiome. Other studies support this scenario where small sample sizes where applied, as the 201 CRESS DNA viruses isolates found associated to faeces of two capybaras (*Hydrochoerus hydrochaeris*) [100] and the potentially novel virus genomes described in 10 specimens of fur seals in Brazil (*Arctocephalus* sp.) [102].

Although the nucleotide sequences reported in this study do not comprise full genomes, this initial characterization contributes to the knowledge of the viral populations that occur in wild animals from South America and has identified potential novel viruses that may be of interest for future studies. This is the first study to use high-throughput sequencing to explore the viral diversity of southern hemisphere wild animals. The findings presented here are expected to help to understand how viral infections in wild animals may impact the health of birds’ population and its potential as sources of viruses which may potentially infect other animal species.

## Figures and Tables

**Table 1:** Adenovirus-like contig ID identified from pool 2 and used for phylogenetic analyses with their respective pairwise identities.

Pool Number	Contig ID	GenBank Accession Number	Virus Name	Closely Related Virus Type	Pairwise Identity (Hexon aa)	Pairwise Identity (DNA Polymerase aa)
**2**	k119 1050	MN025530	Amniota adenovirus 1	*Duck atadenovirus A* (NP_044710.1)	64.5 %.	41.9 %
**2**	k119 2350	MN025529	Psittacine adenovirus 3	Psittacine adenovirus 3 (YP_009112724.1)	97.6 %	---
**2**	k119 2155	MN025529	Psittacine adenovirus 3	Psittacine adenovirus 3 (YP009112716.1)	----	90.2 %
**2**	k119 380	MN153802	Southern psittacara leucophthalmus aviadenovirus	*Fowl aviadenovirus A* (AP_000410.1)	---	61.7 %
**2**	NODE 39	MN153802	Southern psittacara leucophthalmus aviadenovirus	Northern Aplomado falcon adenovirus (AAV90966.1)	73.9 %	---

**Table 2:** Anellovirus-like contig ID identified from pool 1 and used for phylogenetic analyses with their respective pairwise identities.

Pool Number	Contig ID	GenBank Accession Number	Virus Name	Closely Related Virus Type	Pairwise Identity (ORF nt)
1	k119 16753	MN175605	Chicken anemia virus	Chicken anemia virus (JQ308213.1)	98.8%.
1	k119 6992	MN175606	Avian gyrovirus 2	Avian gyrovirus 2 (KX708510.1)	98.8%.
1	k119 6843	MN175607	Gyrovirus GyV3	Gyrovirus GyV3 (MG366592.1)	99.4%
1	NODE 177	MN175608	Brazilian bird anellovirus type 1	Giant panda anellovirus (MF327552.1)	54.6%,
1	NODE 986	MN175609	Brazilian bird anellovirus type 2	Giant panda anellovirus (MF327552.1)	50.7%
1	NODE 1090	MN175610	Brazilian bird anellovirus type 3	Giant panda anellovirus (MF327552.1)	51.7%

**Table 3:** Parvovirus-like contig ID identified from pool 1 and 2 used for phylogenetic analyses with their respective pairwise identities.

Pool Number	Contig ID	GenBank Accession Number	Virus Name	Closely Related Virus Type	Pairwise Identity (NS1 aa)
1	k119 1463	MN175612	Avian chapparvovirus	Turkey parvovirus TP1-2012/HUN (AHF54687.1)	45.3%
2	k119 15398	MN175613	Psittacara leucophthalmus chapparvovirus	Turkey parvovirus TP1-2012/HUN (AHF54687.1)	44.9%
1	k119 1997	MN175614	Avian adeno-associated virus isolate BR_DF	Adeno-associated virus (YP_009552823.1))	42.70%

## Supplementary Materials

The following are available online at https://www.mdpi.com/1999-4915/11/9/803/s1, Table S1: Accession numbers pool1; Table S2: Accession numbers pool2; Table S3: Sequences used in phylogenetic trees.
